# Hemorrhage from the Extraction of a Tooth

**Published:** 1852-10

**Authors:** S. A. Saltonstall

**Affiliations:** Columbus, Mi.


### ARTICLE IX.

*Hemorrhage from the Extraction of a Tooth.* By S. A. Saltonstall, D. D. S., Columbus, Mi.

Messrs. Editors:—Believing it to be quite common to report cases of interest in whatever department of our profession they may occur, I wish to communicate, in as few words as possible, the particulars of an extraordinary case of hemorrhage, which followed the extraction of the first bicuspid tooth, on the right side of the upper jaw.

The subject was Mr. B——, of this place, a member of one of our best families, and a young man of superior attainments, and one of whom much of usefulness was anticipated in the future.

I report the case at his own request as he has recently returned from New York with the title of M. D.

After removing the tooth and using a pledget of cotton saturated with alcohol and pulverized alum, the bleeding, though profuse at the time of the application, ceased, and the patient left my office. Late in the evening the orifice began to emit a copious discharge of blood, and I was sent for to visit him at his father's residence. I used the cork and cotton, which checked, for the time, the flow of blood. The compression was ordered to be continued until the healing process should indicate the safety of removing it. On the second morning, about day-light, I was sent for with the word, "mas John gwine to die." I then used the pure nitrate of silver, which afforded only temporary relief. Finding that this would not do, I used sulphuric acid, first protecting the teeth with beeswax; this failed. I then proposed to apply the actual cautery in the usual way, which was objected to by the consulting physician, who argued that upon its removal, it would bring away with it the coagulum, and only serve to increase the hemorrhage. I began to think that my career as dental surgeon was to end very speedily. The father of the patient was now considerably alarmed, and said to me, "you must do something." At this moment an idea occurred to me that might probably succeed. I mentioned it, and all concurred that it would certainly do; the young man consented to submit to it. I took a piece of pure silver plate, and cut it in shape to fit between the teeth and cover the lips of the orifice about the eighth of an inch on each side. This was bent to fit the parts, and heated to a white heat, and suddenly applied to the place, where it remained for several days. When it was removed, the coagulum came away with it. The orifice was examined, and a very delicate covering, resembling tissue paper, had formed over it.

The success of this operation is mainly attributable to the firmness and presence of mind manifested by the patient. I took my position immediately in front of him, with an instrument bent the right shape to hold the silver, and held it in my left hand; then with an ordinary mouth blow-pipe and a spirit lamp, I applied the heat until the silver was sufficiently hot, while the patient held a napkin firmly over the orifice. At a signal understood by him, he removed the napkin, and I applied the red-hot silver, which arrested effectually the hemorrhage.

